# Weighted Sampling with Frequency-Aware Spatial Attention for Imbalanced Image Classification

**DOI:** 10.3390/jimaging12070293

**Published:** 2026-07-01

**Authors:** Shiqi Zhang, Peng Li

**Affiliations:** 1 School of Mathematics and Statistics, Beijing Institute of Technology, Beijing 100081, China; 2 Department of Earth and Planetary Sciences, Stanford University, Stanford, CA 94305, USA; pli6@stanford.edu

**Keywords:** frequency attention, weighted sampler, hybrid neural network, imbalanced classification

## Abstract

Class imbalance remains a critical challenge in image classification, where underrepresented classes often receive insufficient training attention and exhibit poor recognition performance. In this study, we propose a hybrid framework that combines weighted sampling with frequency-aware spatial attention (WSFSA) to address class imbalance at both the data and feature levels. The weighted sampler improves the training exposure of minority classes, while the frequency-aware spatial attention module incorporates class-frequency information into spatial attention to enhance discriminative feature responses for underrepresented classes. We evaluate the proposed method on four MedMNIST benchmarks, DermaMNIST, BloodMNIST, OrganCMNIST, and DermaMNIST-224, using a ResNet-18 backbone. Results show that WSFSA provides the clearest benefit on the severely imbalanced DermaMNIST and DermaMNIST-224 datasets, performing comparably to the strongest baseline methods while showing particular benefits under severe class imbalance. On OrganCMNIST, WSFSA provides moderate gains, while on BloodMNIST, where the imbalance effect is weaker, all methods perform similarly. Per-class analysis further shows that WSFSA improves sensitivity for several minority or difficult classes while maintaining high specificity across most classes. These findings suggest that combining sampling-level and feature-level rebalancing is a practical strategy for improving class-balanced recognition, particularly under severe class imbalance.

## 1. Introduction

### 1.1. Overall Deep Learning Methods for Image Classification

The task of image classification serves as a fundamental application of deep neural networks (DNNs), with broad use across numerous downstream tasks such as object segmentation [1,2,3], anomaly detection [4,5,6], and multi-class classification [7,8,9]. The technique of image classification has been widely adopted across diverse applications, including autonomous driving, medical imaging, remote sensing, manufacturing, agriculture, etc. Despite the remarkable progress of deep learning in computer vision and imaging, class imbalance remains a critical challenge. In real-world applications, class imbalance arises naturally from the inherent rarity or uneven occurrence of certain conditions or phenomena. However, these underrepresented classes can be critical for understanding the underlying problem and may carry important practical significance. For example, in medical imaging, datasets often contain thousands of normal scans but only a limited number of samples are depicted as rare cancers, uncommon lesions, or early-stage diseases [10,11], and these limited samples are our target of interests that we want to study. Similar patterns appear across multiple domains, such as industrial defect detection [12,13], environmental monitoring [14], and even disaster prediction [15,16], where abnormal events are by nature infrequent but very important to identify accurately.

A wide range of deep learning approaches have been proposed to address the problem of imbalance classification. Here we broadly group the methods into data-level, algorithm-level, and feature-level strategies. Data-level methods, such as oversampling [17,18], undersampling [19,20], and class-balanced sampling [21], directly modify the training distribution to equalize class occurrence. These techniques are straightforward, but excessive oversampling can lead to overfitting, while undersampling may discard valuable information from majority classes. Meanwhile, algorithm-level strategies tackle imbalance by modifying the learning objective. The example methods include class-weighted cross-entropy [22], focal loss [23,24], and cost-sensitive optimization [25,26]. Such methods can emphasize minority classes during training, but they often introduce additional hyper-parameters and can destabilize optimization. Moreover, feature-level approaches aim to enhance the model’s representational power for rare classes, often through attention mechanisms [27,28,29], feature reweighting [30], or contrastive learning [31,32,33]. These methods adaptively focus on discriminative features but typically rely on spatial or channel saliency without explicitly considering the statistical frequency of each class. Here we will introduce in detail the methods in each category.

### 1.2. Data-Level Strategies for Imbalanced Learning

Most of the data-level methods are developed to mitigate class imbalance by operating either on the data or the learning objective. Data-level strategies aim to rebalance the class distribution in the training set. Common techniques include oversampling of minority-class samples [17,18], undersampling of majority-class samples [19], and synthetic data generation (e.g., SMOTE or GAN-based augmentation) [34,35]. These methods ensure that minority classes are sufficiently represented during training, which can improve the model’s sensitivity to rare events. However, oversampling can lead to overfitting due to repeated exposure to the same minority samples, while undersampling may discard valuable information from majority classes. Weighted sampling schemes, which assign probabilities inversely proportional to class frequency, provide a compromise by emphasizing minority classes without fully discarding majority data.

### 1.3. Algorithm-Level Strategies for Imbalanced Learning

Algorithm-level strategies modify the loss function or optimization objective to emphasize the contribution of minority classes. Examples include class-weighted cross-entropy [22], focal loss [23,24] and cost-sensitive learning [25,26], all of which amplify the gradient contributions of underrepresented classes. While effective in some scenarios, these approaches can introduce additional hyper-parameters and sometimes destabilize training, particularly when combined with data-level methods. In practice, careful tuning is required to balance minority-class emphasis with overall model stability.

Although both data-level and algorithm-level approaches have shown promise, they often operate independently of the model’s feature representation. As a result, minority-class features may still be underrepresented in the learned latent space, especially in complex medical imaging tasks with subtle or localized pathologies. This limitation motivates hybrid approaches that integrate feature-level adaptation, such as attention mechanisms, with data-level rebalancing.

### 1.4. Attention Mechanisms for Imbalanced Classification

Attention mechanisms have become a fundamental component in modern deep learning architectures, enabling models to focus selectively on informative regions or features while suppressing irrelevant background information. In the context of imbalanced classification, attention provides an effective way to amplify the representation of minority classes without explicitly altering the data distribution. Early works primarily adopted spatial and channel-wise attention to enhance feature discrimination [36,37]. Spatial attention highlights localized regions that are critical for classification decisions, which are especially beneficial in medical imaging where pathological features may occupy small or subtle regions [38,39]. Channel attention, on the other hand, assigns adaptive weights to feature channels, allowing the network to prioritize class-relevant feature maps [40,41]. Popular frameworks such as the Squeeze-and-Excitation (SE) block [42], CBAM (Convolutional Block Attention Module) [43], and self-attention mechanisms in Vision Transformers (ViTs) [44] have demonstrated improved robustness and interpretability under moderate-imbalance conditions.

However, most existing attention modules are based on feature reweighting. They enhance features purely based on activation strength or spatial saliency, without considering how frequently each class appears in the dataset. As a result, these mechanisms may still favor majority-class patterns, especially when the model learns biased representations during training. Some recent studies have attempted to introduce class-aware attention [45] or reweighting schemes [46], but these approaches often rely on external supervision or predefined priors rather than leveraging class-frequency information directly.

### 1.5. Hybrid and Frequency-Aware Approaches

Recent studies suggest that data-level or algorithm-level strategies alone may not fully address class imbalance, especially in medical imaging, where minority-class features can be subtle and localized. Hybrid approaches that combine multiple levels of rebalancing therefore have been proposed [47,48]. Such methods typically integrate data-level sampling with feature-level adaptation, enabling the model to both see rare classes more frequently and allocate representational capacity to their distinctive features. Several recent studies have explored class-aware or cost-sensitive attention mechanisms, demonstrating improved performance on long-tailed or imbalanced datasets. However, many of these approaches either require external supervision to define class importance or rely on static heuristics that do not adapt dynamically during training. Furthermore, most prior work focuses on either feature-level adaptation or sampling strategies individually, leaving the potential synergy between the two largely unexplored.

### 1.6. Scope of the Work

Motivated by the above observations, our work develops a unified framework that combines a weighted sampling with frequency-aware spatial attention scheme (WSFSA). Our frequency-aware spatial attention module (FSA) embeds class-frequency information into the attention process, allowing the model to focus adaptively on features from minority classes. Combined with a weighted sampler (WS) that balances training data exposure, this framework unifies rebalancing and frequency-guided feature learning to overcome key limitations of existing imbalance mitigation techniques. This design simultaneously addresses sample-level exposure and feature-level emphasis, providing a complementary and interpretable solution to the challenges of imbalanced image classification.

The structure of this paper is organized as follows. Section 1 presents the introduction and background work on class imbalance mitigation using deep learning method. Section 2 introduces the mathematical foundation of proposed framework, including the frequency-aware spatial attention module and the weighted sampling strategy, as well as the experimental setup, datasets, and evaluation protocols used to assess the proposed method. Section 3 reports and discusses the experimental results, highlighting the effectiveness of the proposed approach compared with existing techniques. Finally, Section 4 concludes the paper with a summary of key findings and potential directions for future research.

## 2. Materials and Methods

**Figure 1:**
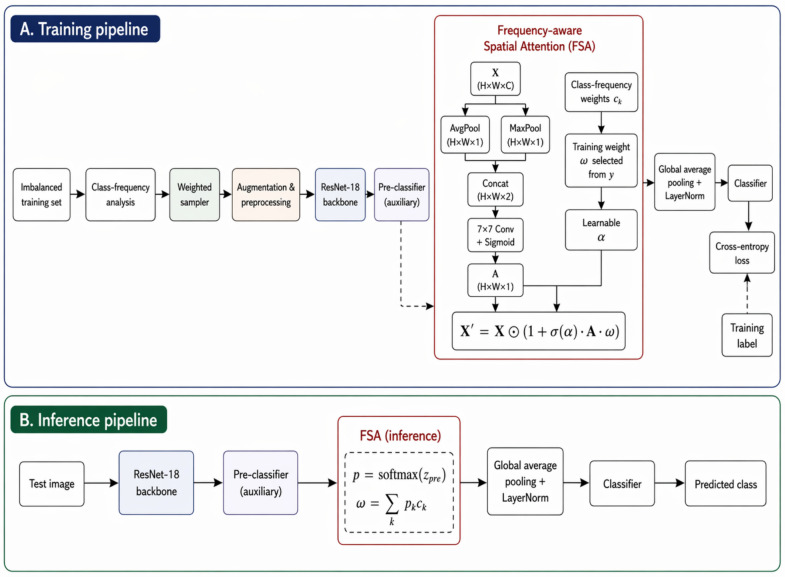
Overall workflow of the proposed method.

In this section, we present the proposed framework for class-imbalanced image classification, which combines frequency-aware spatial attention with a weighted sampling scheme to address both feature- and data-level imbalance. The overall pipeline is illustrated in Figure 1.

### 2.1. Overview

Our framework is built on a ResNet-18 backbone pretrained on ImageNet. To mitigate class imbalance, we integrate two complementary components that operate at different stages of the learning pipeline. The first component is WS (data-level rebalancing), where a square-root inverse-frequency sampler selects training samples with probability proportional to $1/\sqrt{f_{c}}$. This increases minority-class exposure during training without discarding majority-class data. The second component is FSA (feature-level rebalancing). FSA generates a spatial attention map from channel-wise average pooling and max pooling, and then modulates the backbone feature maps using both local spatial activation and class-frequency weights, defined as ${\left(1/\left(f_{c}+\epsilon \right)\right)}^{\beta }$. During training, frequency weights are assigned using ground-truth labels, whereas during inference, an expected frequency weight is estimated from the softmax output of a lightweight pre-classifier. A learnable residual gating parameter α controls the modulation strength and supports stable gradient flow. Together, weighted sampling and FSA form a hybrid rebalancing mechanism that addresses imbalance at both the sampling-distribution and feature-representation levels, improving minority-class sensitivity while preserving overall discriminative performance.

### 2.2. Weighted Sampling

Let the training dataset be

(1) $$D={\left\{\left(x_{i},y_{i}\right)\right\}}_{i=1}^{N},\;y_{i}\in \left\{1,\ldots ,C\right\}$$

where *N* is the total number of samples and *C* is the number of classes. Let $n_{c}$ denote the number of samples belonging to class *c*. To mitigate class imbalance, we assign a sampling weight to each class that is inversely proportional to the square root of its frequency. Specifically, the weight for class *c* is defined as

(2) $$w_{c}=\frac{1}{\sqrt{n_{c}+\epsilon }}$$

where ϵ>0 denotes a small positive constant introduced to avoid division by zero. Each sample *i* inherits the weight of its corresponding class:

(3) $$w_{i}=w_{y_{i}}$$

The sampling probability of sample *i* is obtained by normalizing its weight over all samples:

(4) $$\pi_{i}=\frac{w_{i}}{\sum_{j=1}^{N}w_{j}}$$

where $\pi_{i}$ denotes the probability of sampling sample *i*. The class-level sampling probability P(c) is the aggregate probability that a randomly drawn sample belongs to class *c*:

(5) $$P\left(c\right)=\sum_{i:y_{i}=c}\pi_{i}$$

Substituting $\pi_{i}$ from above, the expected sampling probability for class *c* becomes

(6) $$P\left(c\right)=\frac{n_{c}w_{c}}{\sum_{k=1}^{C}n_{k}w_{k}}$$

This formulation increases the likelihood of selecting samples from rare classes, while preventing overcompensation compared to fully inverse-frequency sampling. These weights are normalized, ensuring that minority-class samples are more likely to be selected during training, while reducing the risk of overfitting associated with naive oversampling.

### 2.3. Frequency-Aware Spatial Attention

The FSA module enhances feature-level representations for underrepresented classes. Given a feature map $X\in R^{B\times C\times H\times W}$, we first compute spatial attention using both average pooling and max pooling along the channel dimension:

(7) $$A_{\mathrm{spatial}}=\sigma \left(\mathrm{Conv}2D\left(\left[\mathrm{AvgPool}(X),\mathrm{MaxPool}(X)\right]\right)\right)$$

where σ(·) denotes the sigmoid activation function. To incorporate class-frequency information, we define a frequency weight for each class:

(8) $$a_{c}=\frac{1/f_{c}^{\beta }}{\max_{k}\left(1/f_{k}^{\beta }\right)}$$

where $f_{c}$ denotes the empirical frequency of class *c*, and β>0 controls the strength of the frequency correction. The attention-modulated feature map is then computed as

(9) $$X^{\prime }=X⊙\left(1+\alpha \cdot A_{\mathrm{spatial}}\cdot a_{c}\right)$$

where α is a learnable parameter that controls the overall contribution of the attention mechanism, and ⊙ denotes element-wise multiplication. This formulation ensures that minority-class features receive proportionally higher attention, thereby improving their discriminative representation.

### 2.4. Weighted Sampling with Frequency-Aware Spatial Attention

The FSA module is inserted after the final convolutional layer of the ResNet backbone. During training, the WS module selects batches with class-balanced exposure, while the FSA module adaptively emphasizes spatial regions relevant to underrepresented classes. The resulting feature maps are then flattened and passed through fully connected layers for classification. Optionally, a logit-level bias can be added to further correct for class imbalance.

The expected contribution of class *c* to the model per training step can be written as

(10) $$E\left[Y_{c}\right]\propto \pi_{c}a_{c}M_{c}=\frac{f_{c}^{-\beta_{s}}}{\sum_{k=1}^{C}f_{k}^{-\beta_{s}}}\cdot \frac{f_{c}^{-\beta_{a}}}{\max_{k}f_{k}^{-\beta_{a}}}\cdot M_{c}$$

where $f_{c}$ is the empirical frequency of class *c*, $\beta_{s},\beta_{a}>0$ control the strength of sampling and attention reweighting, respectively, $\pi_{c}$ is the expected sampling probability of class *c*, $W_{c}$ is the frequency weight for attention, and $M_{c}$ is the spatial attention map for class *c*. This formulation shows how sampling frequency and feature attention are jointly used to prioritize rare classes.

By jointly leveraging data-level sampling and frequency-aware feature modulation, the proposed framework provides a complementary and interpretable approach to imbalanced image classification. The WS module ensures sufficient exposure to minority classes, while FSA amplifies their feature representation in the network, addressing limitations of prior attention-only or sampling-only strategies.

### 2.5. Experiments Design

In this section, we evaluate the proposed WSFSA method on class-imbalanced image classification.

#### 2.5.1. Dataset

We evaluate the proposed method on four datasets from the MedMNIST v2 collection [49], covering three imaging modalities and different levels of class imbalance. The datasets were deliberately selected to span a range of imbalance factors, from approximately 10× to 58×, enabling evaluation under mild, moderate, and severe class imbalance settings. All datasets use the official predefined training, validation, and test splits to ensure reproducibility. For the three standard-resolution datasets, the original images are 28×28 pixels and are resized to 96×96 pixels to provide sufficient spatial resolution for convolutional feature extraction. In addition, we include DermaMNIST-224, a higher-resolution version of our most imbalanced benchmark, to further evaluate the generalizability of the proposed method under a more realistic image-resolution setting.

**Figure 2:**
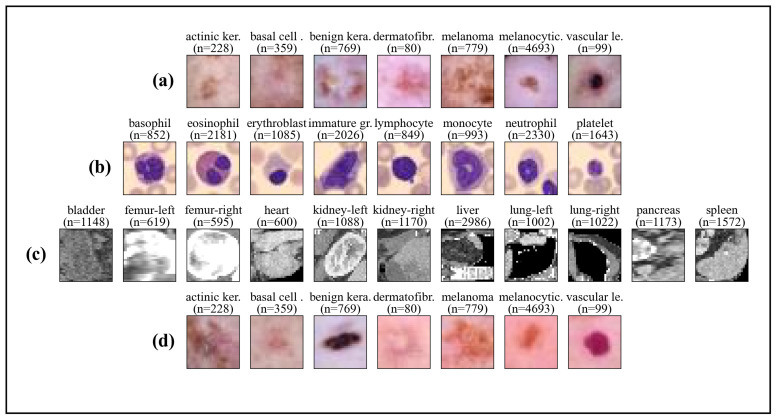
Illustration of images and labels of four datasets: (**a**) DermaMNIST, (**b**) BloodMNIST, (**c**) OrganCMNIST, and (**d**) DermaMNIST-224.

**Figure 3:**
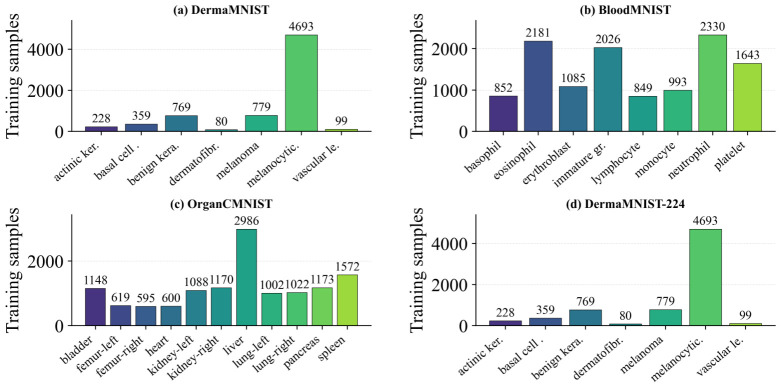
Histograms of data samples from four datasets: (**a**) DermaMNIST, (**b**) BloodMNIST, (**c**) OrganCMNIST, and (**d**) DermaMNIST-224.

DermaMNIST is a dermatoscopic image classification benchmark comprising 10,015 images across seven diagnostic categories (Figure 2a): actinic keratoses (n=228), basal cell carcinoma (n=359), benign keratosis (n=769), dermatofibroma (n=80), melanoma (n=779), melanocytic nevi (n=4693), and vascular lesions (n=99). This dataset exhibits severe class imbalance, with an imbalance factor of approximately 58× (Figure 3a). The train/validation/test split is 7007/1003/2005.

BloodMNIST comprises 17,092 microscopy images of blood cells across eight classes (Figure 2b): basophil, eosinophil, erythroblast, immature granulocyte, lymphocyte, monocyte, neutrophil, and platelet. The imbalance factor is approximately 10× (Figure 3b), and the train/validation/test split is 11,959/1712/3421. This dataset represents the mildest imbalance among the three, allowing us to assess whether the proposed method remains effective under moderate class imbalance.

OrganCMNIST contains 23,660 coronal-view abdominal CT images from 11 organ classes (Figure 2c): bladder, femur-left, femur-right, heart, kidney-left, kidney-right, liver, lung-left, lung-right, pancreas, and spleen. The imbalance factor is approximately 13× (Figure 3c), and the train/validation/test split is 13,000/2392/8268. This dataset introduces additional classification difficulty because several anatomically symmetric organ pairs, such as the left and right femur or kidney, have similar visual appearances at low resolution.

DermaMNIST-224 is the higher-resolution version of DermaMNIST (Figure 2d), containing the same images, diagnostic categories, class distribution (Figure 3d), and train/validation/test split. The main difference is that images are provided at 224×224 pixels, allowing us to evaluate the proposed method under a higher-resolution setting while retaining the same severe class imbalance of approximately 58×.

Together, these datasets cover dermatoscopy, computed tomography, and microscopy, with imbalance factors ranging from 10× to 58× and class counts ranging from 7 to 11. They provide a diverse evaluation setting for assessing the robustness and generalizability of the proposed method across different medical imaging conditions.

#### 2.5.2. Network Backbone

We adopt ResNet-18 [50] pretrained on ImageNet as the feature extraction backbone. The final average pooling and fully connected layers are removed, yielding a convolutional feature extractor that produces spatial feature maps F of dimension 512 × H × W, where H and W denote the spatial dimensions after downsampling. For the baseline model (Vanilla), a global average pooling (GAP) layer reduces the feature maps to a 512-dimensional vector, which is then projected to the number of classes via a single linear layer.

#### 2.5.3. Compared Methods

We compare eight configurations to isolate the contribution of each component:

- Vanilla: ResNet-18 baseline trained with cross-entropy loss and uniform random sampling. This serves as the standard reference.
- Focal: Same architecture as Vanilla, but trained with focal loss, ${\left(1-p_{t}\right)}^{\gamma }\cdot \mathrm{CE}$, with γ=2.0. This setting downweights well-classified examples and focuses learning on hard samples.This serves as the benchmark technology used in literature.
- WS: Vanilla architecture trained with a square-root inverse-frequency weighted random sampler, where each sample’s drawing probability is proportional to $1/\sqrt{f_{c}}$. This rebalances the effective training distribution without modifying the loss function.
- CBAM: ResNet-18 augmented with the Convolutional Block Attention Module (CBAM), trained with uniform sampling and cross-entropy loss. This serves as an attention-based baseline for comparison with the proposed FSA module.
- LDAM-DRW: ResNet-18 trained with the Label-Distribution-Aware Margin loss and deferred reweighting strategy. This serves as a representative loss-level method for long-tailed and imbalanced classification.
- LogitAdjust: Vanilla architecture trained with cross-entropy loss and uniform random sampling, followed by post hoc logit adjustment using class-prior information. This evaluates whether class-prior correction at the output level can improve imbalanced classification.
- FSA: ResNet-18 augmented with the proposed frequency-aware spatial attention module, trained with uniform sampling and cross-entropy loss. Logit bias correction is enabled.
- WSFSA: The combined approach using both WS and FSA. The logit bias is disabled to prevent double correction, since the WS module already adjusts the training distribution.

#### 2.5.4. Training Details

All models are trained for 30 epochs with a batch size of 128 using the AdamW optimizer with an initial learning rate of $10^{-4}$ and a weight decay of 0.05. The learning rate follows a cosine annealing schedule with linear warmup over the first 5 epochs. Cross-entropy loss with label smoothing of 0.1 is used for all methods except Focal, which employs focal loss [23] with γ=2.0.

#### 2.5.5. Evaluation Metrics

In our case, we use the following metrics to provide a comprehensive assessment of the proposed models and their performance under class imbalance:

- Overall accuracy: It defines the fraction of correctly classified samples across all classes. This metric is commonly reported, but it can be dominated by majority classes under imbalanced data distributions.
- Macro accuracy (balanced accuracy): It defines the unweighted mean of per-class recall, giving equal importance to each class regardless of its prevalence.
- Macro F1-score: This represents the unweighted mean of per-class F1-scores, balancing precision and recall across all classes.
- AUC-ROC (macro, one-vs-rest): The area under the receiver operating characteristic curve, averaged across all classes using the one-vs-rest strategy and computed from softmax probabilities.
- Per-class sensitivity (recall): It defines the true-positive rate for each class, measuring the fraction of actual positive samples that are correctly identified. This is our key metric because it reflects the model’s ability to detect each condition.
- Per-class specificity: It represents the true-negative rate for each class, measuring the fraction of actual negative samples that are correctly rejected.

In our case, we emphasize that macro accuracy, macro F1-score, and per-class sensitivity as the primary metrics of interest, since these metrics better reflect performance on minority classes that may be masked by overall accuracy.

## 3. Results

### 3.1. Training Performance

**Figure 4:**
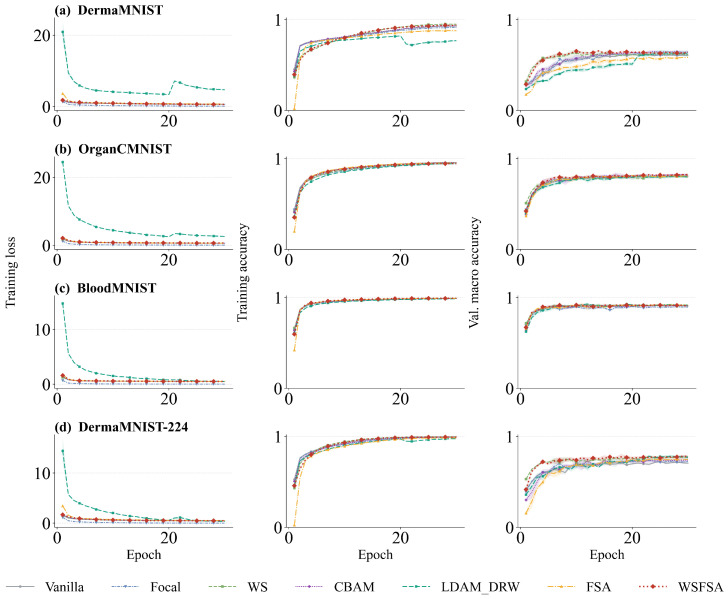
Training curves for all experiments.

We conducted all experiments using the training settings described in Section 2.5.4. To improve statistical reliability, each configuration was independently run three times using different random seeds, and the reported results are summarized using the mean and standard deviation. Figure 4 shows the average training loss, training accuracy, and validation macro accuracy over 30 epochs for DermaMNIST, BloodMNIST, OrganCMNIST, and DermaMNIST-224. Across all datasets, most methods converge within the first 20 epochs, with training loss decreasing rapidly in the early stage and then stabilizing. Focal loss reaches the lowest absolute training loss, although its value is not directly comparable with cross-entropy-based losses due to the focal modulation term. LogitAdjust is applied post hoc to a trained baseline rather than trained as a separate model, so it has no training curve and is omitted from this figure.

### 3.2. Summary Metrics

**Figure 5:**
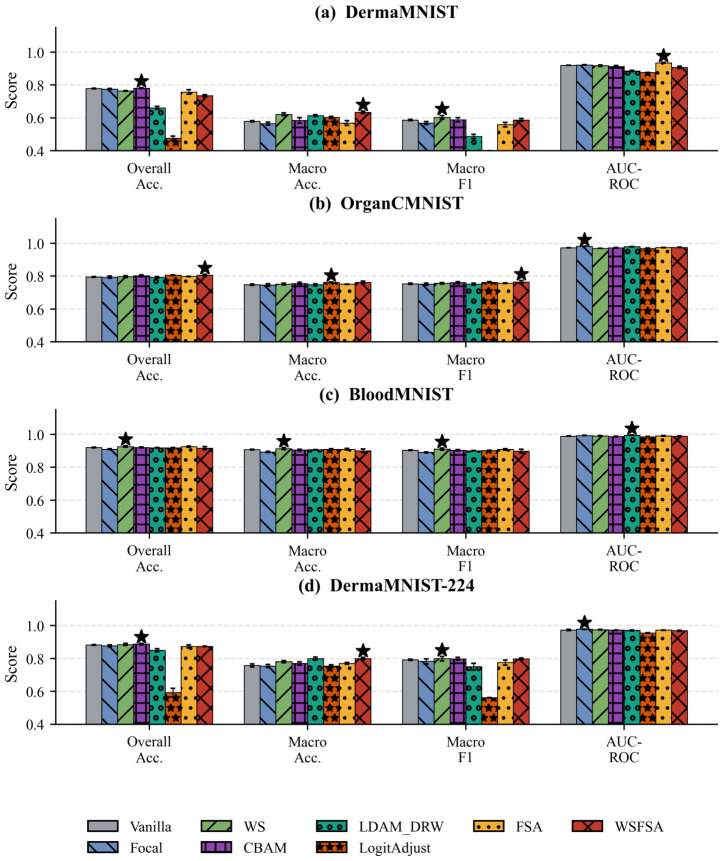
Summary of the statistical metrics for all experiments. The black star marker above a bar denotes the best-performing method for that evaluation metric.

Figure 5 and Table 1 present the overall test-set performance of all eight methods on DermaMNIST, BloodMNIST, OrganCMNIST, and DermaMNIST-224. On DermaMNIST, CBAM achieves the highest overall accuracy (0.781±0.003), while WSFSA achieves the best macro accuracy (0.633±0.007). This represents a +5.4 percentage point (pp) improvement over Vanilla in imbalance-aware accuracy metrics. FSA achieves the highest AUC-ROC (0.935±0.003), suggesting strong discriminative ability across operating thresholds. The gap between overall accuracy and macro accuracy for Vanilla (0.779 vs. 0.579) indicates that standard training remains biased toward majority classes under severe imbalance.

On BloodMNIST, WS achieves the best performance across most metrics, with an overall accuracy of 0.926±0.004, macro accuracy of 0.914±0.006, and macro F1 of 0.910±0.006, while LDAM-DRW achieves the highest AUC-ROC of 0.995±0.000. The smaller performance differences among methods suggest that rebalancing strategies provide less benefit when class imbalance is relatively mild. FSA and WSFSA remain competitive, but neither surpasses the best-performing baseline on this dataset.

On OrganCMNIST, WSFSA achieves the best overall accuracy (0.806±0.005) and macro F1 (0.765±0.009), and LogitAdjust achieves the best macro accuracy (0.762±0.008). Compared with Vanilla, WSFSA improves overall accuracy by +1.1 pp, macro accuracy by +1.4 pp, and macro F1 by +1.2 pp. Focal loss achieves the highest AUC-ROC (0.981±0.000), but its macro accuracy and macro F1 are lower than those of WSFSA. Overall, these results show that WSFSA provides the clearest benefit on DermaMNIST and DermaMNIST-224, with moderate gains on OrganCMNIST, while remaining competitive on BloodMNIST.

On DermaMNIST-224, CBAM achieves the highest overall accuracy (0.887±0.005), while WSFSA achieves the best macro accuracy (0.800±0.007). Compared with Vanilla, WSFSA improves macro accuracy by +4.2 pp, showing that the proposed method remains effective under a higher-resolution setting for the most imbalanced benchmark. WS achieves the highest macro F1 (0.799±0.013), while Focal loss achieves the highest AUC-ROC (0.978±0.001). Overall, the DermaMNIST-224 results further support that WSFSA is most beneficial for improving class-balanced performance under severe class imbalance.

#### 3.2.1. Per-Class Sensitivity

**Figure 6:**
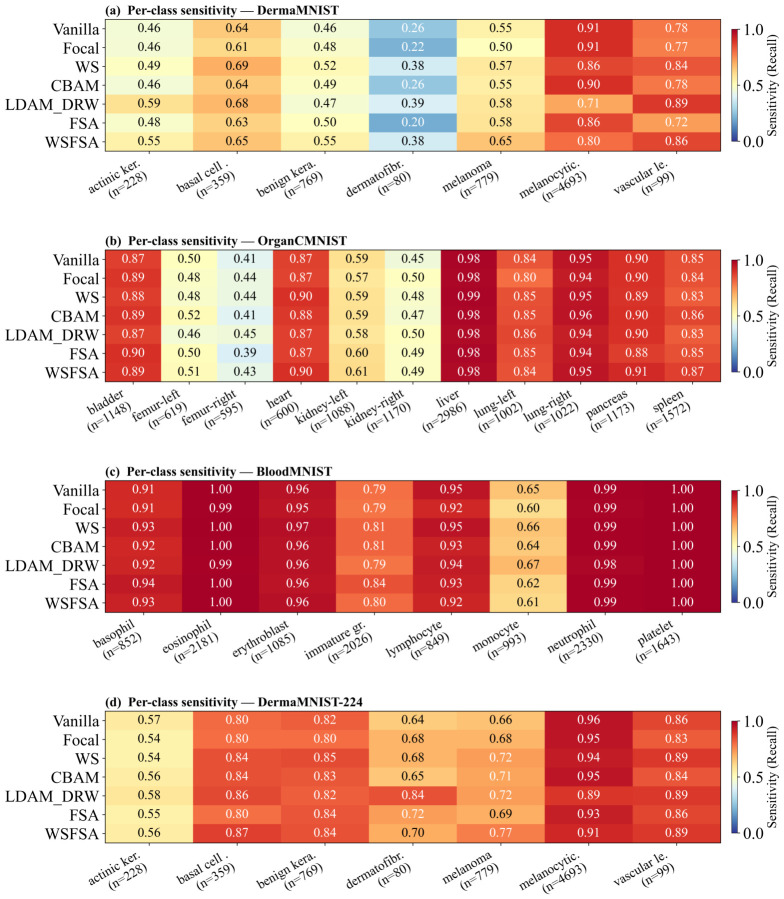
Per-class sensitivity (recall) of all experiments.

Figure 6 shows the average per-class sensitivity (recall) of all methods on DermaMNIST, OrganCMNIST, BloodMNIST, and DermaMNIST-224. On DermaMNIST, WSFSA improves the sensitivity of several minority and clinically important classes. Compared with Vanilla, WSFSA increases sensitivity for actinic keratoses from 0.46 to 0.55, benign keratosis-like lesions from 0.46 to 0.55, dermatofibroma from 0.26 to 0.38, melanoma from 0.55 to 0.65, and vascular lesions from 0.78 to 0.86. WSFSA achieves the highest sensitivity for benign keratosis-like lesions and melanoma, while LDAM-DRW achieves the highest sensitivity for actinic keratoses, dermatofibroma, and vascular lesions. These results indicate that WSFSA improves recall for several minority classes, although the improvement is not uniform across all rare categories.

A similar pattern is observed on DermaMNIST-224, where WSFSA achieves the highest sensitivity for basal cell carcinoma and melanoma, and remains competitive for vascular lesions. However, other methods perform better for some classes, such as LDAM-DRW on dermatofibroma. On OrganCMNIST and BloodMNIST, the per-class sensitivity differences are generally smaller, consistent with the milder class imbalance in these datasets.

On BloodMNIST, all methods achieve high sensitivity for most classes, reflecting the weaker imbalance effect in this dataset. The main difficult class is monocyte, for which LDAM-DRW achieves the highest sensitivity (0.67), followed by WS (0.66) and Vanilla (0.65). WSFSA remains competitive overall, although it does not improve monocyte sensitivity on this dataset. For several other classes, including eosinophil, neutrophil, and platelet, the sensitivities are close to saturation across methods, leaving limited room for improvement.

On OrganCMNIST, WSFSA provides competitive and often improved sensitivity across multiple organs. It achieves the highest sensitivity for kidney-left (0.61), pancreas (0.91), and spleen (0.87), and is tied for the highest sensitivity for heart (0.90). However, other methods perform better for some classes, such as FSA for bladder, CBAM for femur-left and lung-right, LDAM-DRW for femur-right and lung-left, and WS for liver. Overall, the per-class results show that WSFSA improves sensitivity for several minority or difficult classes, particularly on DermaMNIST and OrganCMNIST, while maintaining competitive recall on the less imbalanced BloodMNIST dataset.

A tradeoff is also observed on DermaMNIST: WSFSA reduces sensitivity for the majority class, melanocytic nevi, from 0.91 with Vanilla to 0.80. This reduction is expected for rebalancing-based methods, which shift the model away from majority-class dominance and toward improved recognition of minority classes.

#### 3.2.2. Per-Class Specificity

**Figure 7:**
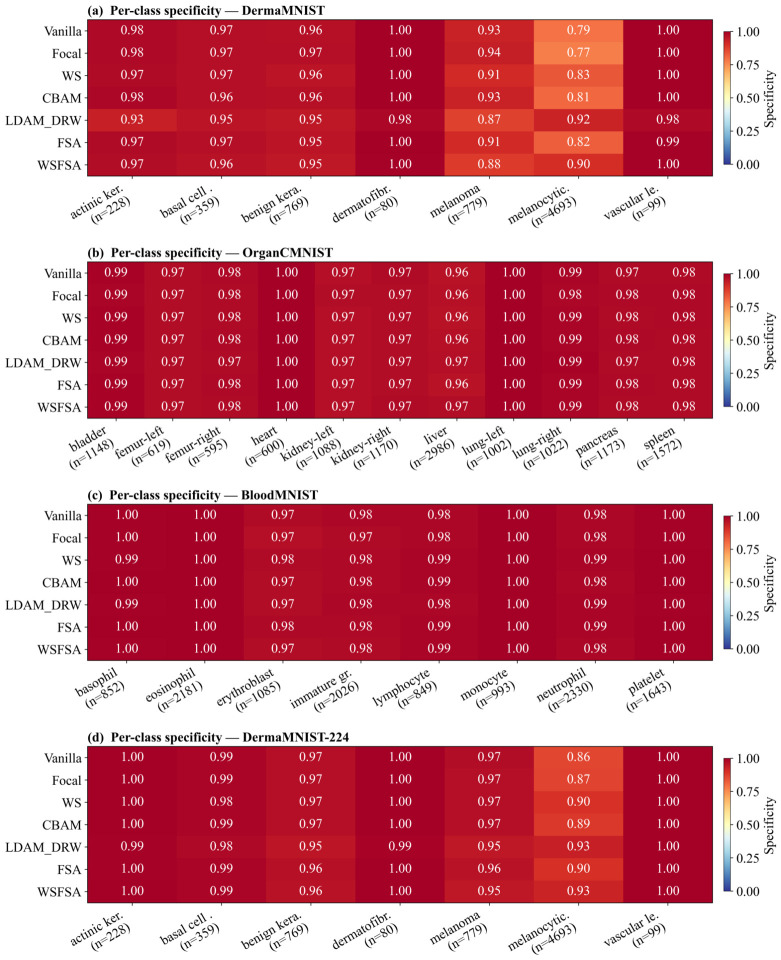
Per-class specificity across four datasets.

Figure 7 presents the per-class specificity of all eight methods on DermaMNIST, OrganCMNIST, BloodMNIST, and DermaMNIST-224. Overall, specificity remains high across the four datasets, indicating that the sensitivity improvements observed in Figure 6 are not mainly caused by indiscriminate over-prediction of minority classes.

On DermaMNIST, most classes maintain specificity above 0.95 across methods. The main exception is the majority class, melanocytic nevi, where Vanilla has a lower specificity of 0.79, suggesting that many non-nevi samples are incorrectly predicted as nevi. WSFSA increases the specificity of this class to 0.90, indicating reduced false positives for the majority class. This improvement is accompanied by modest decreases in specificity for some minority classes, such as melanoma, where WSFSA obtains 0.88 compared with 0.93 for Vanilla.

On DermaMNIST-224, a similar pattern is observed. Specificity remains high for most classes, while WSFSA improves the specificity of melanocytic nevi from 0.86 with Vanilla to 0.93. This suggests that the proposed method also reduces majority-class false positives in the higher-resolution setting.

On BloodMNIST and OrganCMNIST, all methods achieve consistently high specificity, mostly between 0.97 and 1.00. The differences among methods are small, suggesting that the rebalancing strategies do not substantially increase false-positive rates on the less severely imbalanced datasets. Overall, Figure 7 shows that WSFSA improves or maintains competitive sensitivity while preserving high specificity across most classes.

#### 3.2.3. Joint Sensitivity and Specificity Analysis

**Figure 8:**
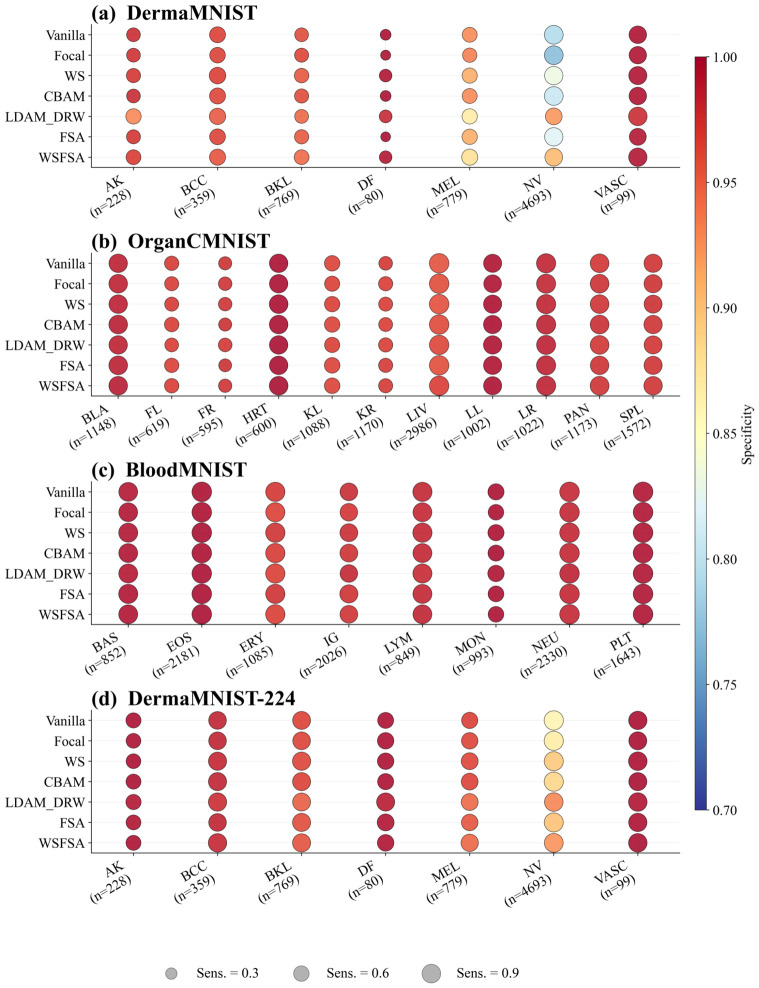
Per-class sensitivity–specificity tradeoff across four benchmarks. Class abbreviations. DermaMNIST: AK, actinic keratoses and intraepithelial carcinoma; BCC, basal cell carcinoma; BKL, benign keratosis-like lesions; DF, dermatofibroma; MEL, melanoma; NV, melanocytic nevi; VASC, vascular lesions. OrganCMNIST: BLA, bladder; FL, femur-left; FR, femur-right; HRT, heart; KL, kidney-left; KR, kidney-right; LIV, liver; LL, lung-left; LR, lung-right; PAN, pancreas; SPL, spleen. BloodMNIST: BAS, basophil; EOS, eosinophil; ERY, erythroblast; IG, immature granulocytes; LYM, lymphocyte; MON, monocyte; NEU, neutrophil; PLT, platelet.

Figure 8 presents a bubble matrix summarizing per-class sensitivity (bubble size) and specificity (color) across all four datasets, allowing simultaneous assessment of both metrics. On DermaMNIST, minority classes such as dermatofibroma (n=80), actinic keratoses (n=228), and vascular lesions (n=99) display smaller bubbles under Vanilla and Focal, reflecting weaker detection of rare conditions. WSFSA enlarges several minority-class bubbles, particularly for actinic keratoses, benign keratosis-like lesions, dermatofibroma, melanoma, and vascular lesions. Meanwhile, the majority class, melanocytic nevi, shifts from lighter coloring under Vanilla to darker red under WSFSA, indicating reduced false positives for this dominant class.

On DermaMNIST-224, a similar pattern is observed in the higher-resolution setting. WSFSA improves sensitivity for several minority or clinically important categories, including basal cell carcinoma and melanoma, while maintaining high specificity across most classes. The specificity of melanocytic nevi also improves under WSFSA, suggesting reduced majority-class false positives.

On BloodMNIST, bubble sizes are uniformly large across most methods and classes, reflecting the weaker imbalance effect in this dataset. The monocyte class remains the most difficult category, with a smaller bubble across methods. WSFSA remains competitive overall, although it does not provide the clearest improvement on this less imbalanced dataset. On OrganCMNIST, several anatomically similar classes, such as femur-left/femur-right and kidney-left/kidney-right, show relatively smaller bubbles across methods, likely due to visual similarity. WSFSA improves or remains competitive for several organs, including kidney-left, heart, pancreas, and spleen.

Overall, Figure 8 shows that WSFSA provides the clearest benefit on the severely imbalanced dermatology datasets, where it improves minority-class sensitivity while preserving high specificity for most classes. The results also highlight an expected tradeoff: rebalancing improves recognition of several underrepresented classes but may reduce sensitivity for the majority class, such as melanocytic nevi.

### 3.3. Ablation and Complexity Analysis

Table 2 reports an ablation on DermaMNIST that isolates the contribution of each component with three random seeds. Starting from the Vanilla baseline, WS improves macro accuracy from 0.579 to 0.622, indicating improved minority-class performance at the expected cost of overall accuracy. WSFSA further improves macro accuracy to 0.633, showing that FSA contributes beyond resampling alone. In contrast, applying FSA without weighted sampling does not improve macro accuracy over the baseline, suggesting that the two components are most effective in combination. Adding a class-prior logit adjustment to WSFSA reduces macro accuracy from 0.633 to 0.605, likely because weighted sampling and FSA already address the imbalance and the additional correction leads to overcompensation. We therefore omit the logit bias from the final model.

To assess deployment feasibility, we measured the parameter count, FLOPs, and per-image inference latency of each model, as reported in Table 3. FSA increases the ResNet-18 backbone by only 1.18% in parameters, mainly due to the auxiliary pre-classifier head, with a negligible FLOP increase and a small latency increase from 0.1834 to 0.1877 ms per image on a Tesla T4. Because the module operates only on the final 7×7 feature map, its computational overhead is minimal and does not substantially affect practical deployment.

We further analyze the error introduced when the inference-time attention weight, $\omega =\sum_{c}\hat{p}\left(c|x\right)w_{c}$, is estimated from the pre-classifier softmax output $\hat{p}$. In isolation, the pre-classifier is weak, with a test accuracy of 0.193 and an expected calibration error of 0.024 after temperature scaling. Its recall is also low for several minority classes, such as melanoma (0.013) and vascular lesions (0.034), indicating a bias toward majority classes. This bias propagates to ω. Because $\hat{p}$ tends to assign most probability mass to majority classes, ω underestimates the target weight for minority-class inputs. Table 4 reports the per-class error, $\delta_{c}=E\left[\omega |y=c\right]-w_{c}$. The error is strongly negative for the rarest classes, such as dermatofibroma (−0.477), and positive for the majority class, melanocytic nevi (+0.401). We also explored calibrating the pre-classifier outputs before computing ω using a validation-fit prior correction, but the optimal correction was negligible because reducing minority-class error introduced larger majority-class error. We therefore treat calibration of the pre-classifier outputs as future work and note the dependence of ω on pre-classifier quality as a limitation.

## 4. Discussion

The results show that WSFSA can improve sensitivity for minority classes without causing a large drop in specificity. This is because WS and FSA help the model in different ways. WS changes which samples the model sees during training, while FSA changes how the model attends to features from different classes. When used together, they give better balanced performance than using either method alone.

There are still two main limitations. First, although we tested the method on four MedMNIST datasets with different imbalance levels, the results are still based on benchmark datasets. Therefore, further validation on larger and more diverse clinical datasets is needed. Second, several settings, including the frequency-weighting exponent β and the logit bias, are selected manually. In particular, β is implemented as a single global value shared across all classes. The relatively low sensitivity of FSA on dermatofibroma suggests that a fixed global β may be insufficient when classes differ substantially in rarity or visual heterogeneity, because it cannot adapt the strength of frequency weighting on a per-class basis.

Future work can improve the method in multiple ways. First, WSFSA could be tested on more real-world datasets with different levels of class imbalance to better understand when it works best. Second, the fixed global frequency-weighting scheme could be replaced with a learnable or class-dependent strategy. For example, the global exponent β could be extended to a per-class parameter $\beta_{c}$, either learned during training or estimated from class statistics. Alternatively, temperature scaling could be applied to the inference-time pre-classifier before computing the expected frequency weight. These extensions may reduce manual tuning and improve robustness for extremely rare or visually heterogeneous classes. Moreover, a more rigorous statistical significance analysis based on a larger number of independent runs would further strengthen the work.

## 5. Conclusions

In this study, we proposed WSFSA, a frequency-aware spatial attention module combined with weighted sampling for class-imbalanced image classification. The method addresses imbalance from two complementary perspectives: WS increases the training exposure of minority classes, while FSA adaptively enhances spatial feature responses for underrepresented classes. Experiments on DermaMNIST, BloodMNIST, OrganCMNIST, and DermaMNIST-224 demonstrate that the effectiveness of WSFSA depends on the severity of class imbalance. On DermaMNIST, where imbalance is most severe, WSFSA achieved the best macro accuracy (0.633±0.007), improving over the Vanilla baseline by 5.4 percentage points. On DermaMNIST-224, WSFSA achieved a macro accuracy of 0.800±0.007, performing competitively with the strongest baseline methods and further demonstrating its effectiveness under a higher-resolution, severely imbalanced setting. On OrganCMNIST, WSFSA achieved the best overall accuracy and macro F1-score, and tied for the best macro accuracy. On BloodMNIST, where class imbalance is relatively mild, all methods performed similarly, and WS achieved the strongest overall class-balanced performance.

The per-class analysis further shows that WSFSA improves sensitivity for several minority or difficult classes, such as actinic keratoses, dermatofibroma, melanoma, and vascular lesions in DermaMNIST, as well as kidney-left, pancreas, and spleen in OrganCMNIST. These gains are achieved while maintaining generally high specificity across classes, indicating that WSFSA does not simply over-predict minority classes. Overall, the results suggest that combining sampling-level and feature-level rebalancing is a practical strategy for improving class-balanced recognition, particularly when the dataset exhibits severe class imbalance.

## Figures and Tables

**Table 1:** Test-set performance across four datasets. Results are reported as mean ± standard deviation. Best mean value per column within each dataset is shown in **bold**.

**(a) DermaMNIST**				
**Method**	**Overall Acc.**	**Macro Acc.**	**Macro F1**	**AUC-ROC**
Vanilla	0.779±0.002	0.579±0.004	0.586±0.004	0.920±0.001
Focal	0.775±0.004	0.565±0.009	0.569±0.009	0.922±0.001
WS	0.764±0.002	0.621±0.008	0.603±0.012	0.917±0.004
CBAM	0.781±0.003	0.585±0.016	0.588±0.013	0.912±0.006
LDAM_DRW	0.661±0.009	0.614±0.005	0.487±0.012	0.886±0.004
LogitAdjust	0.477±0.013	0.602±0.007	0.393±0.007	0.876±0.001
FSA	0.757±0.014	0.568±0.015	0.559±0.014	0.935±0.003
WSFSA	0.735±0.006	0.633±0.007	0.587±0.008	0.908±0.007
**(b) BloodMNIST**				
**Method**	**Overall Acc.**	**Macro Acc.**	**Macro F1**	**AUC-ROC**
Vanilla	0.921±0.002	0.907±0.002	0.904±0.003	0.990±0.001
Focal	0.910±0.003	0.893±0.003	0.891±0.003	0.993±0.000
WS	0.926±0.004	0.914±0.006	0.910±0.006	0.991±0.002
CBAM	0.921±0.003	0.905±0.005	0.904±0.004	0.988±0.002
LDAM_DRW	0.918±0.002	0.907±0.001	0.901±0.001	0.995±0.000
LogitAdjust	0.919±0.002	0.910±0.002	0.903±0.002	0.986±0.001
FSA	0.926±0.004	0.910±0.005	0.909±0.005	0.991±0.001
WSFSA	0.917±0.009	0.901±0.011	0.899±0.011	0.989±0.002
**(c) OrganCMNIST**				
**Method**	**Overall Acc.**	**Macro Acc.**	**Macro F1**	**AUC-ROC**
Vanilla	0.795±0.001	0.748±0.004	0.753±0.003	0.973±0.001
Focal	0.794±0.007	0.746±0.008	0.751±0.007	0.981±0.000
WS	0.797±0.006	0.752±0.006	0.756±0.005	0.969±0.001
CBAM	0.802±0.006	0.756±0.007	0.760±0.007	0.973±0.001
LDAM_DRW	0.793±0.005	0.747±0.007	0.751±0.007	0.979±0.001
LogitAdjust	0.805±0.001	0.762±0.003	0.763±0.003	0.969±0.001
FSA	0.799±0.001	0.751±0.001	0.757±0.002	0.974±0.001
WSFSA	0.806±0.005	0.762±0.008	0.765±0.009	0.975±0.001
**(d) DermaMNIST-224**				
**Method**	**Overall Acc.**	**Macro Acc.**	**Macro F1**	**AUC-ROC**
Vanilla	0.882±0.002	0.758±0.010	0.793±0.003	0.973±0.003
Focal	0.877±0.006	0.753±0.009	0.782±0.015	0.978±0.001
WS	0.885±0.006	0.781±0.006	0.799±0.013	0.975±0.002
CBAM	0.887±0.005	0.770±0.009	0.798±0.008	0.972±0.001
LDAM_DRW	0.850±0.010	0.799±0.009	0.750±0.021	0.971±0.003
LogitAdjust	0.593±0.026	0.754±0.006	0.562±0.001	0.956±0.001
FSA	0.873±0.009	0.770±0.006	0.776±0.015	0.973±0.001
WSFSA	0.874±0.002	0.800±0.007	0.797±0.006	0.969±0.002

**Table 2:** Ablation on DermaMNIST isolating WS, FSA, and the FSA logit bias. Results are reported as mean ± standard deviation over the evaluated seeds. Best mean value per column is shown in **bold**.

Configuration	Overall Acc.	Macro Acc.	Macro F1	AUC-ROC
Vanilla (baseline)	0.779±0.002	0.579±0.005	0.584±0.003	0.920±0.000
WS	0.763±0.002	0.622±0.009	0.601±0.014	0.918±0.005
FSA (no logit bias)	0.769±0.003	0.589±0.005	0.581±0.004	0.935±0.003
FSA	0.749±0.009	0.559±0.009	0.549±0.000	0.933±0.000
WSFSA	0.732±0.006	0.633±0.007	0.590±0.009	0.904±0.006
WSFSA + logit bias	0.734±0.019	0.605±0.016	0.566±0.002	0.905±0.010

**Table 3:** Model complexity and inference efficiency comparison.

Model	Params	Params (M)	GFLOPs	Latency (ms)	Std.	Batch Size	Image Size	Hardware
ResNet-18 (Vanilla)	11,180,103	11.1801	0.6699	0.1834	0.0013	64	96	Tesla T4
ResNet-18 + CBAM	11,213,514	11.2135	0.6700	0.1863	0.0013	64	96	Tesla T4
ResNet-18 + FSA	11,311,794	11.3118	0.6702	0.1877	0.0010	64	96	Tesla T4

**Table 4:** Per-class approximation error of the inference-time attention weight on DermaMNIST. $w_{c}$ denotes the max-normalized inverse-frequency target weight, and $\delta_{c}=E\left[\omega |y=c\right]-w_{c}$ denotes the estimation error.

Class	Train *n*	$w_{c}$	$\delta_{c}$
Dermatofibroma	80	1.000	−0.477
Vascular lesions	99	0.899	−0.367
Actinic keratoses & intraepithelial carcinoma	228	0.592	−0.045
Basal cell carcinoma	359	0.472	+0.069
Benign keratosis-like lesions	769	0.323	+0.200
Melanoma	779	0.320	+0.199
Melanocytic nevi	4693	0.131	+0.401

## Data Availability

The data presented in this study are openly available in the MedMNIST v2 collection at https://medmnist.com (accessed on 28 June 2026).

## Abbreviations

The following abbreviations are used in this manuscript:

DNN	Deep neural network
FSA	Frequency-aware spatial attention
pp	Percentage point
WS	Weighted sampling
WSFSA	Weighted sampling with frequency-aware spatial attention
